# Cancer Drug Resistance: Targeting Proliferation or Programmed Cell Death

**DOI:** 10.3390/cells13050388

**Published:** 2024-02-23

**Authors:** Elena V. Sazonova, Maria A. Yapryntseva, Nikolay V. Pervushin, Roman I. Tsvetcov, Boris Zhivotovsky, Gelina S. Kopeina

**Affiliations:** 1 Engelhardt Institute of Molecular Biology, Russian Academy of Sciences, 119991 Moscow, Russia; sazonova_82@mail.ru (E.V.S.); smariaal@mail.ru (M.A.Y.); rhododendron.nick@mail.ru (N.V.P.); tsvetcovroman7@gmail.com (R.I.T.); 2 Faculty of Medicine, MV Lomonosov Moscow State University, 119991 Moscow, Russia; 3 Division of Toxicology, Institute of Environmental Medicine, Karolinska Institute, P.O. Box 210, 17177 Stockholm, Sweden

**Keywords:** chemotherapy, resistance, proliferation, cell death

## Abstract

The development of resistance to chemotherapy is one of the main problems for effective cancer treatment. Drug resistance may result from disturbances in two important physiological processes—cell proliferation and cell death. Importantly, both processes characterize alterations in cell metabolism, the level of which is often measured using MTT/MTS assays. To examine resistance to chemotherapy, different cancer cell lines are usually used for the in vitro modulation of developing resistance. However, after the creation of resistant cell lines, researchers often have difficulty in starting investigations of the mechanisms of insensitivity. In the first stage, researchers should address the question of whether the drug resistance results from a depression of cell proliferation or an inhibition of cell death. To simplify the choice of research strategy, we have suggested a combination of different approaches which reveal the actual mechanism. This combination includes rapid and high-throughput methods such as the MTS test, the LIVE/DEAD assay, real-time cell metabolic analysis, and Western blotting. To create chemoresistant tumor cells, we used four different cancer cell lines of various origins and utilized the most clinically relevant pulse-selection approach. Applying a set of methodological approaches, we demonstrated that three of them were more capable of modulating proliferation to avoid the cytostatic effects of anti-cancer drugs. At the same time, one of the studied cell lines developed resistance to cell death, overcoming the cytotoxic action.

## 1. Introduction

A tumor is a pathological formation that appears because of the accumulation of mutations in the genome of cells that promote their uncontrolled division and evasion of programmed cell death (PCD). The biology of tumor cells is based on the selection of the most malignant clones [1]. As a result, such clones acquire a number of adaptive advantages, including increased cell proliferation and resistance to PCD [2]. Notably, although the different approaches of anti-cancer therapy target these features of tumor cells, deficiencies in PCD can neutralize all attempts [3,4,5,6,7]. The initiation of PCD and cell cycle arrest promote the activation of different extra- and intracellular molecular networks that lead to the inhibition of cancer progression [3,8]. Thus, DNA-damaging chemotherapeutic agents can stop cell division and trigger the most well-known mode of PCD—apoptosis. However, the administration of many anticancer drugs results in the development of drug resistance, which has many aspects [9,10]. For example, cancer cells are able to avoid PCD (including apoptosis) and immune control, accelerate cell division, pump out anti-cancer drugs, and alter the metabolism to adjust to oxygen and nutrient restriction, etc. Importantly, many studies have aimed to identify the mechanisms of resistance, but the first question that needs to be addressed is what this resistance means: overcoming cytotoxic drug activity (appearance of defects in PCD), or the ability to escape cytostatic action (modulation of proliferation and metabolism) in tumors. It is important to understand which of these effects would predominate.

Cancer cell lines are a very common model for studying the mechanisms of therapeutic resistance [11,12]. Importantly, the first question which arises for researchers is the choice of the most suitable model for the development of resistance. In general, there are two known in vitro alternatives: (1) the short treatment of cancer cells with low concentrations of a drug followed by a drug-free interval (pulse-selection method); and (2) the long-term (up to 6 months) incubation of tumor cells with high doses of anticancer agents [13,14]. Both models have their own characteristics. The first approach mimics the conditions of chemotherapy of cancer patients when cancer cells reside in the presence of low drug concentrations during relatively short periods and have sufficient time for recovery. This is exactly how patients undergo several cycles of chemotherapy. Thus, this approach is considered to be a clinically relevant model. In this case, based on a comparison of IC50 values, an increase in resistance to anticancer agents is not too high (1.5–5-fold) [14,15,16,17]. Importantly, these data are in good consistency with resistant index for cancer cells derived from patients [13]. The second model of resistance development enables the creation of cell lines with a high level of resistance. In this case, cancer cells grow in the presence of high drug concentrations over time, which promotes the survival of the most malignant clones. However, this approach does not correspond to the conditions when patients undergo anticancer therapy and may lead to artificial results being obtained. Nevertheless, both approaches are widely used in different investigations [11,14,15,18,19]. At the same time, despite the huge number of studies devoted to this topic, there is still a lack of understanding regarding which set of methods would allow the most complete information to be obtained about the mechanisms of resistance development [13].

Based on previously published results [20], we have chosen four different cell lines to study the mechanisms of chemotherapeutic resistance: A549 (lung carcinoma), U1810 (lung carcinoma), SKOV3 (ovarian cancer), and SW620 (colon cancer). Cisplatin-resistant cells were obtained from these lines using the step-by-step incubation of cancer cells with chemotherapeutic drug, namely, the pulse-selection approach as the most clinically relevant model, since we aimed to differentiate the cytotoxic and cytostatic effects of cisplatin in the conditions mimicking cycles of chemotherapy in patients. The used cell lines originated from various types of tumors with different mutation statuses of the p53 protein, displaying the most illustrative changes upon treatment with cytotoxic and cytostatic drugs. Cisplatin was used as the agent for resistance formation because platinum-based drugs are the first-line treatment of solid tumors. However, cisplatin has many side effects such as a lack of selectivity, high systemic toxicity, and the development of drug resistance [21].

IC50 values evaluated using the MTS test allowed us to assess the decreased sensitivity to cisplatin in resistant cells. However, it remained unclear whether the resistance was due to the eluding of cytotoxic action or the modulation of the proliferation and cellular metabolism. To solve this question, we used the LIVE/DEAD assay to evaluate the numbers of both viable and dead cells. The clonogenic and metabolic assay helped us to estimate the proliferation rate and metabolic status of cells, and additionally confirmed the results of the LIVE/DEAD test. Using Western blot analysis, we estimated molecular mechanisms of cisplatin action in parental and resistant cells. In this way, we developed methodological guidelines to show the drug resistance origin. Finally, we demonstrated that cisplatin resistance in three cell lines firstly aroused cell cycle perturbation and resulted in the avoidance of cell death in the fourth cell line.

## 2. Materials and Methods

### 2.1. Cell Culture and Treatments

The lung adenocarcinoma cell line A549 (ATCC, CCL-185), lung large cell carcinoma U1810 (CVCL D054), the colorectal adenocarcinoma cell line SW620 (ATCC, CCL-227), and the ovarian adenocarcinoma cell line SKOV3 (ATCC, HTB-77) were kindly provided by the Department of Toxicology, Karolinska Institute (Stockholm, Sweden). Cells were routinely checked for mycoplasma. The cells were grown in Dulbecco’s modified Eagle medium (DMEM) with 4.5 g/L glucose (Gibco, Waltham, MA, USA) supplemented with antibiotic-antimycotic (Gibco), and 10% fetal bovine serum (Gibco). Cells were grown in a CO_2_ incubator (5% CO_2_) at 37 °C and re-cultured every 2–3 days using 0.15% trypsin solution (Gibco). Throughout the experiments, the cells at the logarithmic growth phase were treated with cisplatin (Teva, Tel Aviv, Israel) for 72 h. To block the apoptosis induced by this compound, a selective inhibitor of caspases and, subsequently, apoptosis Q-VD-Oph (Selleck Chemicals LLC, Houston, TX, USA), was added for 1 h prior to cisplatin.

### 2.2. Development of Drug-Resistant Cell Lines

A stepwise dose incremental strategy was used to obtain cisplatin-resistant cell lines A549, U1810, SKOV3, and SW620 [15]. Briefly, the A549-, U1810-, SKOV3-, and SW620-resistant sublines were selected upon constant exposure of the parental wild-type (wt) cells to cisplatin (Teva, Tel Aviv, Israel) in a stepwise dose incremental strategy.

For each cell line, using the MTS assay, the half-maximal inhibitory concentration (IC50) of cisplatin was calculated. The cell lines were treated with increases in the dosage of the drug, ranging from IC10 (A549 and U1810—2 μM; SKOV3 and SW620—1 μM), IC20 (A549 and U1810—4 μM; SKOV3 and SW620—2 μM), IC40 (A549 and U1810—8 μM; SKOV3 and SW620—4 μM), to IC50. Cells were incubated for 48 h with each concentration of the drug. Following the respective drug treatments, the viable cells were transferred in drug-free medium for a period of 3–5 days. After these cells were obtained, they were no longer cultured in the presence of the drug. These cells were used for the MTS assay to assess the IC50 value post exposure.

### 2.3. Drug Sensitivity Assay

Briefly, the used cell lines were grown at a concentration of 1.5 × 10^3^ cells/well in 96-well plates. The cells were incubated overnight in the CO_2_ incubator. The cells were subsequently treated with serial dilutions of cisplatin or vehicle control for 72 h. An MTS assay was performed, according to the manufacturer’s instructions. Briefly, 20 µL MTS (CellTiter 96 AQueous One Solution Cell Proliferation Assay, Promega, Madison, WI, USA) was added to the cells and incubated for 4 h at 37 °C. Following MTS incubation, the spectrophotometric absorbance of the samples was estimated using a VarioScan Flash microplate reader (Thermo Fisher Scientific, Waltham, MA, USA) at 480 nm with a reference wavelength of 630 nm. Cells without the drug were used as controls. The percentage of viable cells was calculated using the following equation: mean optical density (OD) of the experiment/mean OD of the control ×100. The IC50 values in each case were calculated using regression analysis in GraphPad Prism 8 software (GraphPad Software, San Diego, CA, USA), and are expressed as an average of triplicate experiments. The resistance index (RI) was calculated by the ratio of the IC50 of resistant cell lines to wild-type cell lines.

### 2.4. LIVE/DEAD Assay for Mammalian Cells

Cells were cultured into flat-bottom 96-well plates (Nunc), with 1500 cells per well in full growth medium overnight. Next, the cells were treated with cisplatin, topotecan (a topoisomerase inhibitor used to treat ovarian, lung, and many other cancer types), and Q-VD for 72 h, as described above. After treatment, the cells were stained with the LIVE/DEAD Viability/Cytotoxicity Kit for mammalian cells (Thermo Fisher Scientific) by diluting 2 μM of calcein-AM and 4 μM of ethidium homodimer-1 (EthD-1) in Dulbecco’s phosphate-buffered saline (DPBS, PanEco, Moscow, Russia).

For the quantitative evaluation of viable and dead cell numbers, VarioScan Flash microplate reader was used. The Calcein-AM signal from viable cells was detected 15 min after dye addition using excitation and emission filters at 485 nm and at 517 nm. The EthD-1 signal from dead cells was detected 45 min after dye addition using excitation and emission filters at 530 nm and at 617 nm. Empty wells were used for background signal estimation; cells incubated with 70% ethanol for 30 min were used as a positive control for EthD-1 staining.

### 2.5. Western Blotting

After harvesting with 0.1% trypsin-EDTA solution, the cells were centrifuged (1000 rcf, 4 min, +4 °C), washed with DPBS (PanEco), and centrifuged again. The pellet was incubated for lysis in radioimmunoprecipitation assay (RIPA) buffer (25 mM Tris-HCl, 150 mM NaCl, 1% NP-40, 0.5% sodium deoxycholate) containing a Halt protease inhibitor cocktail (Thermo Fisher Scientific) and phosphatase inhibitor cocktail (Sigma-Aldrich, Darmstadt, Germany) for 15 min on ice. After centrifugation (15,000 rcf, 15 min, +4 °C), the supernatant was taken to determine the protein concentration, and to use for Western blotting. The protein concentration was assessed using a BCA Protein Assay Kit (Thermo Fisher Scientific). Protein samples were mixed with a Laemmli’s buffer and heated at 95 °C for 5 min. Proteins were separated using the TGX Stain-Free™ FastCast™ acrylamide kit (Bio-Rad, Hercules, CA, USA). Next, proteins were moved to a nitrocellulose membrane at 110 V for 110 min in a wet tank transfer system (Bio-Rad, Hercules, CA, USA). To block non-specific protein binding sites, the membranes were incubated in a 5% solution of nonfat milk powder in TBST buffer (20 mM Tris, 150 mM NaCl, 0.025% Tween 20, pH 7.4) for 45 min at room temperature. After that, the membranes were washed in TBST (4 × 5 min) and incubated with primary antibody at 4 °C overnight. The membranes were washed in TBST (4 × 5 min) and incubated with secondary antibody in 2.5% nonfat milk powder solution for 1 h at room temperature. After washing membranes in TBST, a signal was induced using Clarity Western ECL substrate (Bio-Rad). The ChemiDoc XRS+ System (Bio-Rad) was used for chemiluminescence detection and image analysis. The following primary antibodies were used at the indicated dilution: rabbit monoclonal to MDR1/ABCB1 (clone D3H1Q) diluted 1:1000 (12683), rabbit polyclonal phospho-SAPK/JNK (Thr183/Tyr185) diluted 1:1000 (9251), rabbit polyclonal SAPK/JNK diluted 1:1000 (9252), rabbit polyclonal phospho-p38 (Thr180/Tyr182) diluted 1:1000 (9211), rabbit polyclonal p38 diluted 1:1000 (9212), rabbit polyclonal GPX4 diluted 1:1000 (52455), rabbit polyclonal to cleaved CASP3 diluted 1:1000 (9661), rabbit monoclonal to phospho-histone H2AX (Ser139) (clone 20E3) (9718), rabbit monoclonal to GAPDH (clone 14C10) diluted 1:3000 (2118) (all from Cell Signaling Technology),rabbit polyclonal to PARP1 diluted 1:1000 (ab137653), mouse monoclonal to SQSTM1/p62 (clone 2C11) diluted 1:1000 (ab56416), rabbit polyclonal to MAP1LC3B diluted 1:3000 (ab51520) (all from Abcam), and mouse monoclonal to CASP3 (clone 19/Caspase-3/CPP32) diluted 1:1000 (610323, BD Biosciences, San Diego, CA, USA). The original uncropped images of Western blot membranes are provided in the Supplementary Materials.

### 2.6. Clonogenic Assay

The cells were seeded at low confluency (1000 cells per well in 2 mL culture medium) in triplicate in six-well plates. Then, the cells were cultured for 10–14 days. After incubation, the cells were washed with PBS twice, fixed with 4% paraformaldehyde solution in PBS, and stained with 0.5% crystal violet in aqueous solution. Next, the plates were imaged with the ChemiDoc XRS+ System (Bio-Rad) and analyzed using ImageJ software (version 1.53t).

### 2.7. Metabolic Assays

Cells were plated in wells of a 96-well Seahorse microplate using standard culture media. After reaching 70–80% confluence, cells were treated with IC50 of cisplatin and incubated for 24 h. After incubation, cells were washed and then incubated in assay medium for 1–3 h in a non-CO_2_ incubator at 37 °C. The assay medium was DMEM without FBS, phenol red, glycose, and sodium pyruvate supplemented with 2 mM of glutamine for glycolysis evaluation. The same medium supplemented with 1 mM sodium pyruvate and 10 mM glucose was used for respiration evaluation. The oxygen consumption rate (OCR) and extracellular acidification rate (ECAR) were evaluated in real time using the Seahorse XF Extracellular Flux Analyzer (Agilent, Santa Clara, CA, USA). During the respiration evaluation, 1 μM of oligomycin, 1 μM of carbonyl cyanide m-chlorophenyl hydrazone (CCCP), and 1 μM of rotenone/antimycin A1 were added to wells to estimate different parameters of respiratory function. The first three measurements were used for basal respiration. During glycolysis evaluation, 10 mM of D-Glucose, 1 μM of oligomycin, and 50 mM of 2-deoxyglucose were added to wells. The measure of glycolysis, presented as the ECAR, was reached after the addition of saturating amounts of glucose. The data were normalized to the protein content in each well.

### 2.8. Flow Cytometry Analysis of subG1 Population and Cell Death Evaluation

After harvesting, cells were resuspended in DPBS. Ice-cold 70% ethanol was added drop by drop with continuous mixing; then, the samples were incubated for at least 60 min at −20 °C. Before measuring, the cells were centrifuged and resuspended in DPBS. Next, the cells were incubated for 15 min after the addition of propidium iodide (50 μg/mL) and RNase A (100 μg/mL). The cells were analyzed using the FACS Canto II flow cytometer (BD).

### 2.9. Data Processing and Statistical Analysis

Three or more independent repeats were performed for each experiment unless stated otherwise in the figure legend. Calculations of IC50, statistical analysis, and data plotting were performed using GraphPad Prism 8 software (GraphPad Software, San Diego, CA, USA). The differences between experimental groups were analyzed using the two-sided Mann–Whitney U-test and one-way ANOVA with Sidak’s multiple comparison test (ns, non-significant difference; * *p* < 0.05; ** *p* < 0.01; *** *p* < 0.001; **** *p* < 0.0001).

## 3. Results and Discussion

### 3.1. Creation and Characteristics of the Resistant Cell Lines

**Figure 1:**
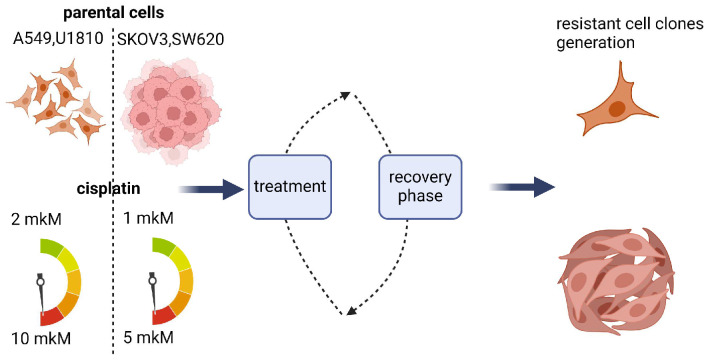
Development of a cisplatin-resistant cell line to study the biological changes leading to resistance. Design for the creation of resistant cell lines (for details, see Section 2).

Plenty of studies aiming to overcome resistance have been conducted to investigate how tumor cells escape death [21,22,23,24]. Less attention has been paid to studying the slowdown of cell proliferation as a way for tumor cells to acquire resistance. To investigate this issue, four cell lines resistant to cisplatin were developed and resistance mechanisms were evaluated using various approaches. To create resistant cancer cells, the IC50 of cisplatin (50% of maximum possible response) was estimated for these model cell lines using the MTS assay. Cisplatin is a widely used chemotherapeutic agent for the treatment of various tumors (intestinal, bladder, head and neck, lung, ovarian, and testicular cancers), which causes DNA crosslinking [21]. The cell lines U1810, A549, SKOV3, and SW620 were subsequently exposed to increasing concentrations (IC10, IC20, IC40, and IC50) of cisplatin for the development of resistance (Figure 1).

**Figure 2:**
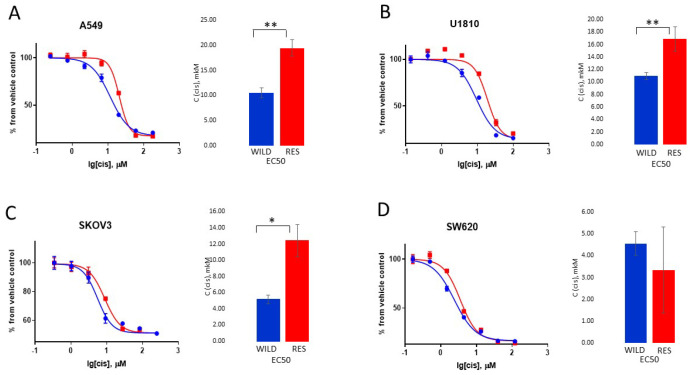
Response of wild-type and cisplatin-resistant cells to treatment with various concentrations of cisplatin. The MTS assay values obtained from wild-type and cisplatin-resistant A549 (**A**) and U1810 (**B**), SKOV3 (**C**), and SW620 (**D**) cells were normalized to the untreated sample. The data are presented as the mean ± standard deviation (N = 3). “WILD”—wild-type cells; “RES”—cells resistant to cisplatin; “cis”—cisplatin; * *p* < 0.05; ** *p* < 0.01.

To avoid excess toxic effects after 3 days of treatment, cells were incubated in the drug-free media for the recovery period required for each cell line. The obtained resistant cell lines—U1810R, A549R, SKOV3R, and SW620R—were assessed for resistance using the MTS assay. A549R, U1810R, and SKOV3R cells demonstrated statistically significant increases in IC50 compared with the wild-type cell line: the resistance index (RI = IC50 wild-type cells/IC50-resistant cells) was 1.6–2.4 (Figure 2A–C, Table S1). These results are consistent with the published data, which reveal 1.5–2-fold increases in IC50 for cisplatin-resistant cells obtained using a similar pulse-selection approach [16,17]. SW620R cells demonstrated an interesting exception (RI = 1) (Figure 2D, Table S1), which can be the result of unusual characteristics of this cell line and a questionable interpretation of the MTS assay that was previously described [16,22,25]. Indeed, Wang et al., using the MTS assay, showed that SW620 cells incubated with a high dose of cisplatin for 6 months were not characterized by any difference in viability in comparison with untreated cells [25].

### 3.2. Cytotoxic and Cytostatic Effects of Cisplatin Treatment

**Figure 3:**
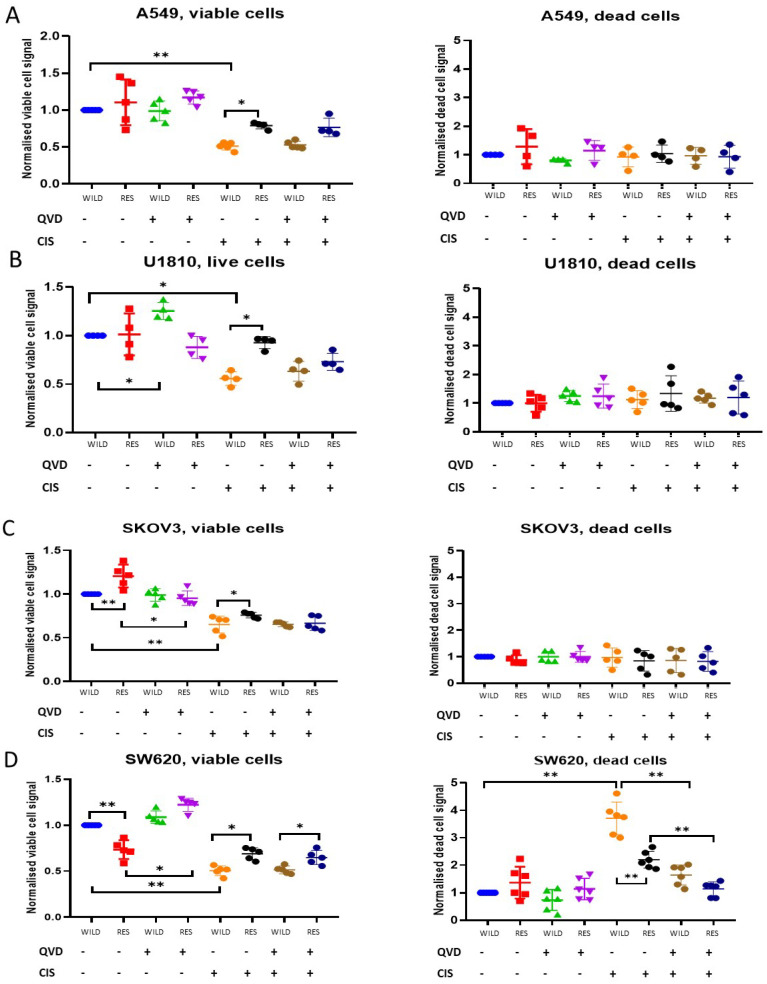
Cytotoxic and cytostatic effects of cisplatin treatment in SW620, SKOV3, A549, and U1810 cell lines according to the LIVE/DEAD assay. Wild-type and cisplatin-resistant A549 (**A**), U1810 (**B**), SKOV3 (**C**), and SW620 (**D**) cells were treated with cisplatin (5 µM for SW620 and SKOV3, 10 µM—for U1810 and A549 cell line) in the presence or absence of 25 μM of Q-VD for 72 h. Calcein-AM (viable cell) and EthD-1 (dead cell) signals were obtained via the LIVE/DEAD assay. The data are normalized to the respective “Contr” samples; the lines and whiskers indicate the mean ± standard deviation (N = 4). “WILD”—wild-type cells; “RES”—cells resistant to cisplatin; “cis”—cisplatin; * *p* < 0.05; ** *p* < 0.01.

The main question for the investigation of developed resistance is whether resistance to cisplatin is exercised through the inhibition of the most common form of PCD: apoptosis. To address this question, wild-type and cisplatin-resistant cell lines were treated with this drug at concentrations corresponding to IC50 values for wild-type cells—5 µM for SW620 and SKOV3, 10 µM for U1810 and A549 cell lines (Table S1)—in the presence or absence of the pan-caspase inhibitor Q-VD (25 μM). We used IC50 for wild-type cells to estimate the cytotoxic and cytostatic effects of cisplatin in equal conditions for both resistant and parental lines. The populations of viable and dead cells were assessed using the LIVE/DEAD assay (Figure 3).

Analysis showed that in contrast to other studied cell lines, cisplatin treatment led to significant increase in the number of dead (EthD-1-positive) SW620 cells versus control (Figure 3D). At the same time, the level of cisplatin-induced death of SW620R cells was statistically decreased in comparison to wild-type (RI = 1.7, Table S1). Simultaneously, the decrease in viable SW620R cells upon the drug treatment was essentially lower compared with wild-type cells. The other cell lines—A549, U1810 and SKOV3—did not demonstrate an increased number of dead cells after cisplatin administration (Figure 3A–C, Table S1). This result highlighted the unique characteristics of SW620 cells and their susceptibility to PCD. These findings revealed that SW620 cells had become resistant to cisplatin, even though the MTS test did not show an increase in IC50 for SW620R cells. These observations point to the importance of using different approaches to estimate the resistance of cancer cells.

Cisplatin-induced decreases in viable wild-type SW620 cells were partially blocked by the addition of Q-VD. At the same time, the number of dead wild-type cells decreased significantly upon combined treatment with cisplatin and pan-caspase inhibitor in comparison to cisplatin alone (Figure 3D). These data revealed that cisplatin induced apoptosis in SW620 cells. Upon the drug treatment, resistant cells demonstrated higher survival rates and the reduced accumulation of dead cells in comparison to the wild type (Figure 3D). The combined treatment of SW620R cells with cisplatin and Q-VD protected them from apoptosis but was not so effective in wild-type cells. These data indicated that the developed resistance of SW620 cells to cisplatin resulted from the perturbation of PCD mechanisms.

For the SKOV3, A549, and U1810 lines, LIVE/DEAD assays demonstrated a situation that was very different from SW620. As mentioned above, the incubation of wild-type cells with cisplatin at the IC50 concentration significantly decreased the number of viable cells, but did not lead to an increase in the population of dead cells (Figure 3A–C, Table S1). It is remarkable that the addition of Q-VD to cisplatin did not increase the number of viable cells, suggesting that cisplatin does not trigger substantial caspase-dependent apoptosis in SKOV3, A549, and U1810 cells. Interestingly, in resistant cell lines, the cisplatin treatment still statistically decreased the number of viable cells. These data indicated that the resistant cells could affect the cell cycle to avoid toxic effect of the drug. Thereby, the proliferation speed of resistant cells was lower than in wild-type populations, resulting in less accumulation of live cells upon cisplatin-induced genotoxic stress.

**Figure 4:**
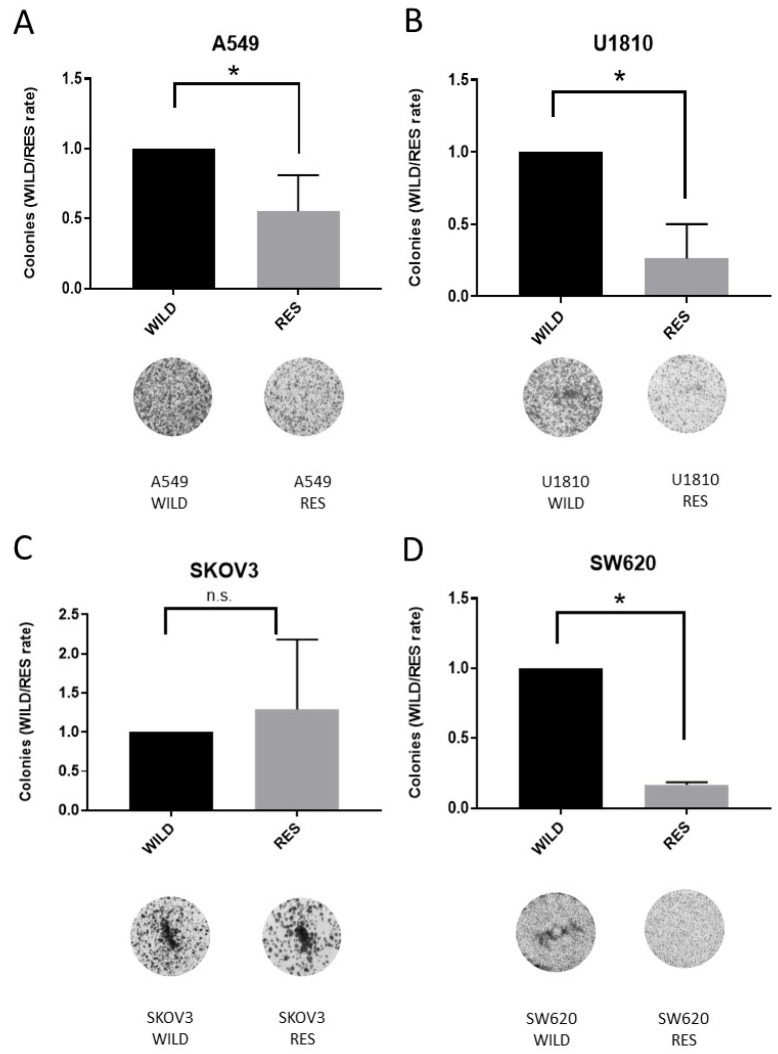
Clonogenic assay of parental and resistant cell lines A549 (**A**), U1810 (**B**), SKOV3 (**C**), and SW620 (**D**). The data are presented as a rate (WILD/RES cells) of the number of colonies, normalized to wild-type cells (N = 4); n.s.—non-significant difference. “WILD”—wild-type cells; “RES”—cells resistant to cisplatin; ns, non-significant difference, * *p* < 0.05.

Based on the LIVE/DEAD test, we hypothesized that there were differences in the growth rate between the wild-type and resistant cell lines. For instance, SW620R demonstrated a significant decrease in the accumulation of the number of live (calcein-AM-positive) cells in the control. To study this point, we used a clonogenic assay that quantifies the number of colonies generated from viable cells [26]. This assay revealed that the wild-type SW620 cells proliferate faster than SW620R (Figure 4).

A similar pattern was observed for U1810 and A549 cell lines, while the clonogenic resource of resistant SKOV3 cells showed the same result as wild-type cells (Figure 4). Importantly, according to the LIVE/DEAD test, SKOV3R cells demonstrated the more prominent accumulation of dead (calcein-AM-positive) cells in comparison with the wt cell line without any treatment. Consistent with this, the clonogenic assay allowed us to conclude that resistant SKOV3 cells surpassed the wild-type cells in their ability to proliferate, generating an increasing number of clones prone to uncontrolled division. The other cell lines—SW620, A549, and U1810—underwent the suppression of cell division during the development of cisplatin resistance. Potentially, this strategy allows cells to repair DNA lesions after long incubation with cisplatin.

Taken together, based on data obtained from the LIVE/DEAD and clonogenic assays, we concluded that resistance of the studied cell lines to cisplatin had different mechanisms. The SW620 cell line became insensitive to the induction of cell death (cytotoxic mechanism) and to the slowdown of cell proliferation (cytostatic mechanism). The resistant U1810, A549, and SKOV3 cell lines were able to modulate the speed of proliferation to avoid the action of cisplatin.

### 3.3. Biochemical Evaluation of Cisplatin Resistance

Cisplatin can trigger various molecular mechanisms of action, including different forms of PCD—apoptosis, necroptosis, ferroptosis, and autophagy [27,28,29,30]. The depression of PCD is a popular strategy by which tumor cells avoid the toxic effects of anti-cancer drugs. Moreover, one of the key mechanisms of resistance is the increased pumping out of the drug from cells through energy-dependent transporters; for example, the MDR1/ABCB1 protein belonging to the ATP-binding cassette transporter superfamily [31,32,33]. This protein is normally expressed in the plasma membranes of various cells with different tissue origins, including the intestinal endothelium, the blood–brain barrier, and adrenal cells [34]. We evaluated the levels of MDR1/ABCB1 and biomarkers of several types of PCD using Western blotting (WB). Notably, because the level of any proteins used as a loading control could be changed during the creation of resistant lines, the level of studied proteins was normalized to the total protein levels for each sample quantified using TGX stain-free gels.

**Figure 5:**
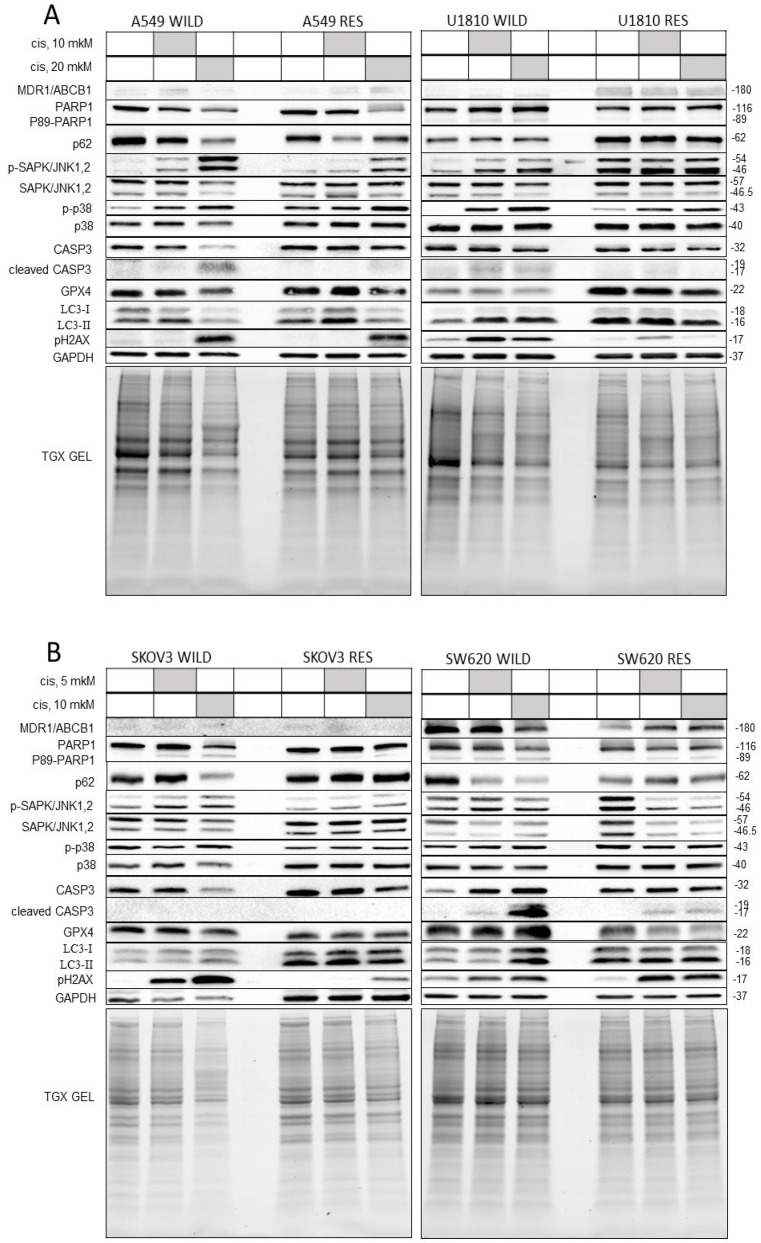
Western blot analysis of protein levels in lysates of wild-type and cisplatin-resistant cells. Wild-type and cisplatin-resistant A549 and U1810 (**A**), SKOV3 and SW620 (**B**) cells were treated with cisplatin (10 and 20 µM for U1810 and A5495; 10 µM for SW620 and SKOV3 cell line) for 72 h. The samples are indicated above; proteins of interest are indicated on the left; molecular weight markers are indicated on the right. “Contr”—control sample; “Cis”—cisplatin. “WILD”—wild-type cells; “RES”—cells resistant to cisplatin.

According to Western blot, significant differences in the levels of MDR1/ABCB1 were only detected between the U1810 wt and U1810R cells, indicating that MDR is not the leading mechanism of resistance for all the studied cell lines (Figure 5 and Figure S2). Consistent with this, the level of phosphorylated histone H2AX—one of the main markers of DNA damage—was decreased in U1810R cells upon cisplatin treatment compared with wild-type cells (Figure 5A and Figure S2A). This confirmed that DNA lesions were more slowly accumulated in the resistant line due to the overexpression of the transporter and the enhanced pumping out of cisplatin.

Next, we assessed the level of the 89 kDa fragment of poly(ADP-ribose) polymerase-1 (PARP1) and the cleaved active form of effector caspase-3 as apoptotic markers. In all resistant cell lines, the accumulation of p89 PARP was less pronounced than in wild-type cells (Figure 5 and Figure S1). Decreased levels of cleaved caspase-3 in SW620R, U1810R, and A549R cells clearly confirmed this result. It was shown that the cleaved form of caspase-3 was not detected in either wild-type or resistant SKOV3 cells. Importantly, the absence of caspase-3 activation in this cell line was described previously [34]. This may be because proapoptotic signal transmission in SKOV3 cells occurs via caspase-7, for which PARP1 is a substrate that is similar to caspase-3 [35,36,37,38]. Notably, the SW620 cell line demonstrated the most prominent accumulation of p89 PARP and the active form of caspase-3, confirming its ability to trigger apoptosis (Figure 5 and Figure S1). According to WB data, cisplatin can induce apoptosis in all studied cell lines. This was the inconsistency observed between the Western blotting and LIVE/DEAD assays. This difference can arise from the fact that WB analysis, unlike the LIVE/DEAD test, allows a signal to be collected from all cells, which makes this test more sensitive. Taken together, the combination of WB and LIVE/DEAD assays indicated that the resistance of all cell lines to cisplatin was developed through the avoidance of cytostatic and cytotoxic mechanisms in different proportions; for example, SW620 cells are more prone to apoptosis induction. For this reason, in the resistant variant of this line, the imbalance of apoptotic mechanisms is more prominent than in the other three cell lines.

One of the main problems complicating the effective treatment of cancer is cross-insensitivity, when drug treatment results in resistance to other medications. To investigate this, we used the SW620 cell line, which was shown in a previous study to be sensitive to cisplatin and topotecan via the induction of apoptosis [20]. Here, we set out to test whether the SW620R cells would possess resistance to topoisomerase I inhibitor topotecan. The responses of wild-type and resistant cells to treatment with topotecan at the IC50 concentration 20 nM (detected previously, see [20]) were examined using flow cytometric analysis of the subG1 population and WB to assess apoptotic markers. The results of the subG1 analysis showed no differences in the accumulation of topotecan-induced subG1 populations between SW620 wt and SW602R cells (Figure S2A). The detection of cleaved forms of caspase-3, p89 PARP1, and phospho-H2AX in cell lysates after incubation with topotecan demonstrated the activation of the apoptotic pathway in wild-type as well as resistant cells (Figure S2B). Therefore, SW620 cells showing resistance to cisplatin remained sensitive to topotecan, providing the opportunity to overcome cisplatin insensitivity.

To investigate the molecular mechanisms of cytotoxic and cytostatic effects of cisplatin, the levels of the following proteins were examined: (1) mitogen-activated protein kinases (MAPK)—c-Jun N-terminal kinases (JNKs) 1/2 and p38—as regulators of the cell cycle and apoptosis; (2) MAP1LC3B-II and SQSTM1/p62—the key players of autophagy; and (3) selenocysteine-containing protein GPX4, which pivotally controls ferroptosis. The last form of PCD is characterized by iron-dependent lipid peroxidation and metabolic constraints [35,36,37].

JNK1/2 and p38—members of the mitogen-activated protein kinase family—are responsive to the regulation of different cellular processes, including cell cycle control, differentiation, and apoptosis. WB analysis of these proteins and their phosphorylated active forms demonstrated that the accumulation of pJNK1/2 decreased in A549R and SKOV3R cell lines (Figure 5 and Figure S1). Moreover, SKOV3R cells additionally demonstrated the decreased phosphorylation of p38 (Figure 5 and Figure S1). Both resistant cell lines accumulated less phosphorylated H2AX. JNK and p38 regulate the phosphorylation of H2AX at Ser139 [39], which facilitates the recruitment of proteins participating in DNA damage detection and repair [40]. We supposed that one of the important mechanisms of resistance to cisplatin of A549 and SKOV3 cells to cisplatin resulted from the decreased activity of these MAPKs. Most likely, the inhibition of JNK1/2 and p38 kinases in SKOV3R cells leads to autophagy activation, which may also play a role in drug resistance [41,42]. This is consistent with the accumulation of a well-known autophagosome marker, MAP1LC3B-II, in SKOV3R cells (Figure 5 and Figure S1). In the other cell lines, we did not observe any significant changes in autophagy markers, suggesting that autophagy did not have an essential contribution to the development of resistance.

Finally, we analyzed the level of GPX4 as a marker of ferroptosis. The results of WB showed that the level of this protein was higher in U1810R cells than in wild-type cells, while we did not detect any differences between wild-type and resistant cells in the cell lines SW620, A549 and SKOV3 (Figure 5 and Figure S1). Cisplatin treatment has been shown to activate ferroptosis [43] and increase GPX4 levels, one of the possible mechanisms of resistance to the toxic effect of this drug in U1810 cell line.

Taken together, biochemical analysis revealed that the cisplatin resistance could lead to disturbances in the different forms of PCD—apoptosis, autophagy, and ferroptosis. However, it should be noted that the suppression of PCD mechanisms was more prominent in the SW620 cell line, indicating its sensitivity to the cytotoxic effect of cisplatin. These results are consistent with the previously obtained data showing that the toxic effects of chemotherapeutic drugs should be divided into cytotoxic and cytostatic [20].

### 3.4. Metabolic Status in Cells Resistant to Cisplatin

Surprisingly, according to WB analysis, the glyceraldehyde-3-phosphate dehydrogenase (GAPDH) level, which is frequently used as a loading control, was lower in SKOV3R compared with wild-type cells (Figure 5 and Figure S1). This could indicate metabolic changes in resistant cells. GAPDH exhibits both dehydrogenase and nitrosylase activities, thereby playing a key role in glycolysis and numerous intracellular processes, such as membrane fusion, microtubule binding, phosphotransferase activity, nuclear RNA export, and DNA replication and repair [44,45]. The baseline metabolism of wild-type and cisplatin-resistant SKOV3, SW620, U1810, and A549 cells was studied by assessing glycolysis and mitochondrial respiration levels using the Seahorse analyzer (Agilent technology, Santa Clara, CA, USA).

**Figure 6:**
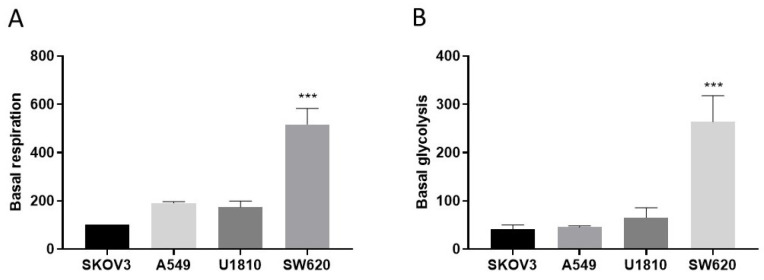
Assessments of the basal respiration rate (**A**) and basal glycolysis rate (**B**) of wild-type A549, U1810, SKOV3, and SW620 cell lines using the Seahorse XF Extracellular Flux Analyzer, measured from 4 independent experiments. One-way ANOVA with Sidak’s multiple comparison test was used for statistical analysis; *** *p* < 0.001.

By measuring the OCR and ECAR in the studied cell lines, we found that wild-type SW620 cells were much more metabolically active than other cell lines, according to tests of respiration and glycolysis (Figure 6).

**Figure 7:**
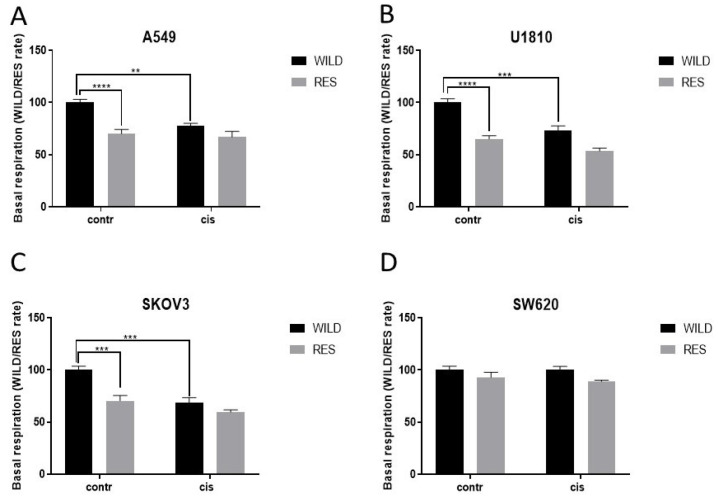
Assessment of the basal respiration rates of A549 (**A**), U1810 (**B**) SKOV3 (**C**), and SW620 (**D**) using the Seahorse XF Extracellular Flux Analyzer. The data were normalized to wild-type cells and presented as the WILD/RES rates (from 3 independent experiments). Two-way ANOVA with Sidak’s multiple comparison test was used for statistical analysis. “WILD”—wild-type cells; “RES”—cells resistant to cisplatin. “Contr”—control sample; “Cis”—cisplatin; ** *p* < 0.01; *** *p* < 0.001; **** *p* < 0.0001.

Based on the assessment of metabolism, resistant SW620 cells did not show any suppression of mitochondrial respiration, but resistant U1810, A549, and SKOV3 cell lines were characterized by a significant decrease in mitochondrial respiration (Figure 7).

Cisplatin treatment led to an essential decrease in respiration in U1810, A549, and SKOV3 wild-type cell lines. In contrast to the wild type, resistant cells of all four lines did not demonstrate some changes in respiration upon cisplatin exposure that confirmed formation of alterations in their metabolism during development of resistance (Figure 7). Similar results were obtained for the basal glycolytic rate (Figure S3). Taken together, these data highlight the engagement of changes in glycolysis and respiration in the formation of resistance.

These data indicated that the development of cisplatin resistance in SW620 cells took a completely different path compared with U1810, A549, and SKOV3 cells. As mentioned above, these three cell lines can escape drug toxicity, firstly through modulation of the cell cycle, which leaves a mark on cell metabolism. The obtained data corroborated some published results. Thus, it was shown that cisplatin-resistant A549 cells are characterized by an increased mass of mitochondria and activation of the proliferation of tumor cells [46]. It was also found that resistance to cisplatin in non-small cell lung cancer cells is associated with an abrogation of cisplatin-induced G2/M cell cycle arrest [47]. In SKOV3, long non-coding RNA LINC00184, which determines cell proliferation, is highly expressed, which could stimulate the development of cisplatin resistance [48]. In contrast, acquired resistance was only able to inhibit PCD in SW620 cells but did not affect respiration. Importantly, the inhibition of apoptosis in cisplatin-resistant colon cancer cells through different mechanisms has been found [49,50], which confirms the data obtained in this study.

## 4. Conclusions

Despite significant advances in cancer treatment, acquired resistance to therapy is one of the key problems of modern oncology [10,32,51]. For a number of the cell lines we used in this study, the resistance to treatment has previously been described [19,25,46,47,49,50,52,53,54,55,56,57]; however, the mechanisms for resistance of U1810 cell line to cisplatin have not been investigated. Many studies in this area have focused on mechanisms showing how tumor cells escape the cytotoxic and/or cytostatic effects of drugs. We used a pulse-selection approach, which mimics cycles of anticancer therapy of patients, to create four resistant cell lines with different origins. Using the set of methods, we demonstrated that the formation of resistance through insensitivity to cell death is not a frequent cause of drug resistance. In three out of four studied cell lines (A549, U1810, and SKOV3), the formation of resistance to cisplatin was mainly dependent on a cytostatic mechanism. Thus, to assess developed resistance, it is important to evaluate not only the sensitivity to cell death, but also the arrest of proliferation, as well as changes in cell metabolism.

Summarizing the obtained results, we can suggest a route to analyze the characteristics of resistant cell lines. At the first stage, it is advisable to use a combination of the MTT/MTS assay and the LIVE/DEAD test in the presence or absence of selective inhibitors of cell death to obtain information on whether resistance has formed through cytotoxic or cytostatic mechanisms. Clonogenic assays can estimate the proliferation rate of resistant cells for the correct interpretation of data obtained from the two above-mentioned approaches. At the second step, it is important and helpful to assess molecular mechanisms of resistance using Western blotting analysis. In the final step, to gain in-depth knowledge concerning changes in cell metabolism, it is beneficial to use different forms of real-time cell metabolic analysis. Using this set of approaches, it is easy to draw conclusions concerning the mechanism of resistance in each particular case.

## Data Availability

The authors have no data to deposit in a repository. The original uncropped images of Western blot membranes are provided in the Supplementary Data.

## Supplementary Materials

The following supporting information can be downloaded at: https://www.mdpi.com/article/10.3390/cells13050388/s1, Table S1. Fold resistance for A549R, U1810R, SKOV3R, and SW620R compared with their parental cell lines according to MTS and LIVE/DEAD tests. Figure S1. Immunoblot quantification of MDR1/ABCB1, P89-PARP1, p62, p-p38, p38 p-SAPK/JNK1,2 (p-JNK), SAPK/JNK1,2 (JNK), caspase 3 (full c3), p19-caspase-3 (cl-c3), LC3-I, LC3-II, GPX4, pH2AX, and GAPDH protein levels normalized to TGX gel A549 and U1810 (A), SKOV3 and SW620 (B) cell lines. The levels of p-p38 and pJNK1/2 were normalized to their non-phosphorylated forms. All values are in relative units. Each boxplot includes values from three independent experiments. Figure S2. Evaluation of the cross-resistance of cisplatin-resistant SW620 cells to topotecan. (A) Sub-G1 analysis of wild-type and cisplatin-resistant SW620 cells treated with 20 nM topotecan for 72 h. Values are the mean (±standard deviation of the mean) of three independent experiments. (B) Wild-type and cisplatin-resistant SW620 cells were treated with 20 nM topotecan for 72 h. Immunoblot analysis using the indicated antibodies is shown. The samples are indicated above; proteins of interest are indicated on the left. “WILD”—wild-type cells; “RES”—cells resistant to cisplatin; “Contr”—control sample; “top”—topotecan. Figure S3. Assessment of the basal glycolysis rate of A549 (A), U1810 (B), SKOV3 (C), and SW620 (D) cell lines using the Seahorse XF Extracellular Flux Analyzer. The data were normalized to wild-type cells and are presented as WILD/RES rates (from 3 independent experiments). Two-way ANOVA with Sidak’s multiple comparison test was used for statistical analysis. “WILD”—wild-type cells; “RES”—cells resistant to cisplatin; “Contr”—control sample; “Cis”—cisplatin. Figure S4. Uncropped immunoblot images of A549 (A), U1810 (B), SKOV3 (C), and SW620 (D) cell lines.
