# Supplementary material for: Cancer Drug Resistance: Targeting Proliferation or Programmed Cell Death
Source: Cells. 2024 Feb 23;13(5):388. doi: 10.3390/cells13050388 (PMC10930385; doi:10.3390/cells13050388)
Supplement: Supplementary file 1 [file cells-13-00388-s001.zip › cells-2843016-supplementary.pdf]

	A549 wt	A549 res	RI	U1810 wt	U1810 res	RI	SKOV3 wt	SKOV3 res	RI	SW620 wt	SW620 res	RI
IC_{50}, µM (MTS)	10,5±1,0	19,44 ±1,63	1,9	10,93 ±0,20	16,88 ±0,39	1,6	5,17±0,54	12,41 ±1,98	2,4	4,56±2,60	3,34±0,70	0,7
normalized dead cell signal (LIVE/DEAD)	1,08±0,16	1,13±0,24	1,0	1,23±0,20	1,33±0,55	1,0	0,97±0,36	0,84±0,39	0,9	3,71±0,65	2,20±0,23	1,7

Table S1. Fold resistance for A549R, U1810R, SKOV3R and SW620R compared with their parental cell lines according to MTS and LIVE/DEAD tests.

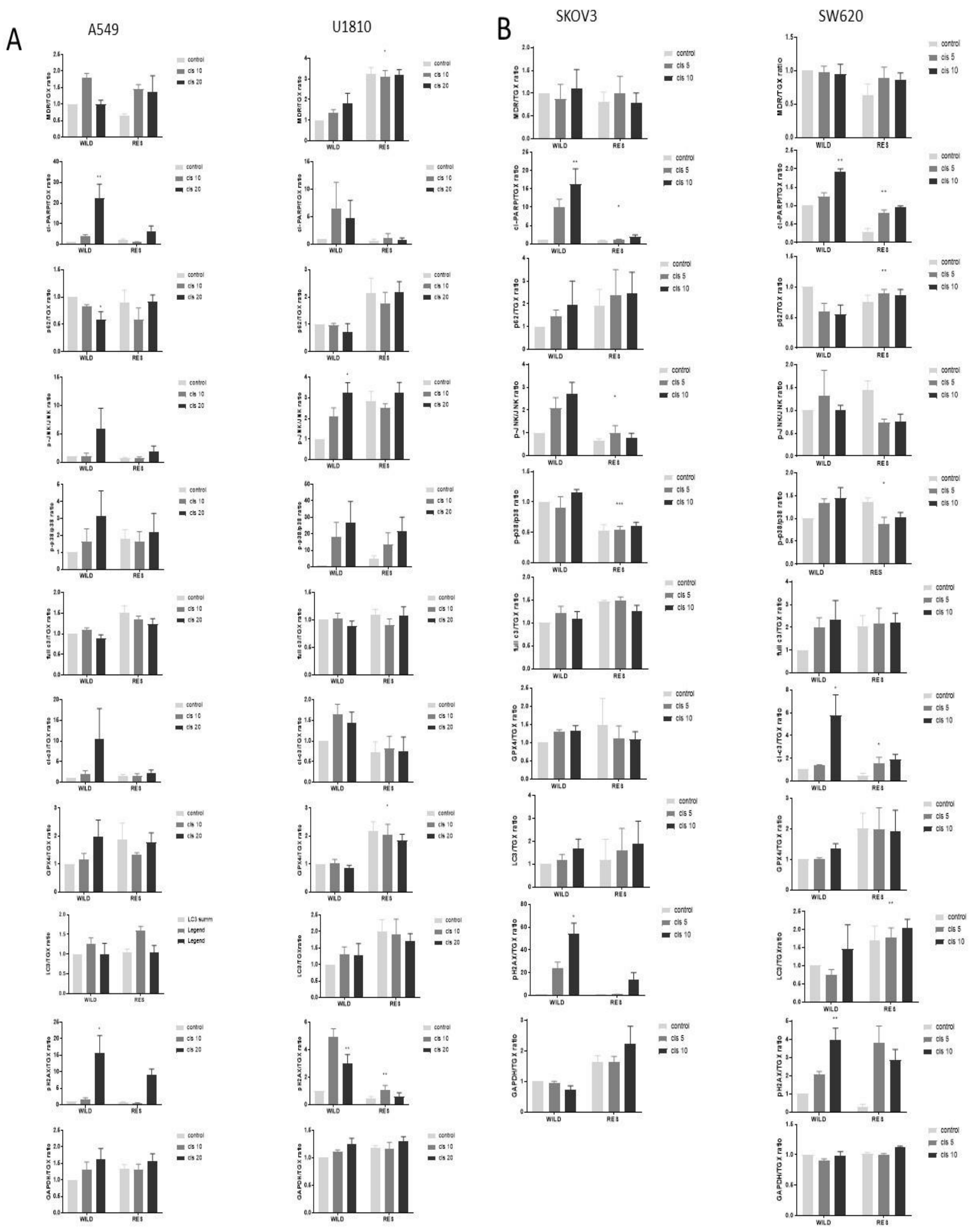

Figure S1. Immunoblot quantification of MDR1/ABCB1, P89-PARP1, p62, p-p38, p38 p-SAPK/JNK1,2 (p-JNK), SAPK/JNK1,2 (JNK), caspase 3 (full c3), p19-caspase-3 (cl-c3), LC3-I, LC3-II, GPX4, pH2AX, GAPDH protein levels normalized to TGX gel A549 and U1810 (A), SKOV3 and SW620 (B) cell lines. The levels of p-p38 and pJNK1/2 were normalized to their non-phosphorylated forms. All values are in relative units. Each boxplot includes values from three independent experiments.

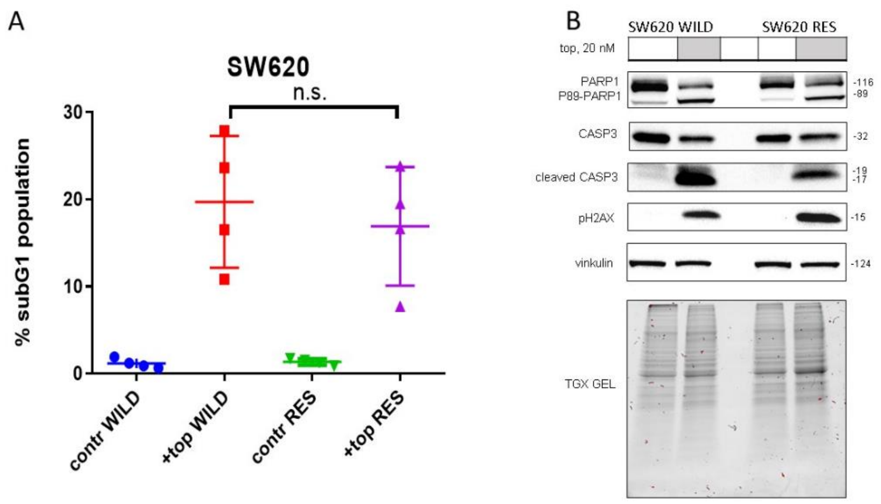

Figure S2. Evaluation of cross-resistance of cisplatin-resistant SW620 cells to topotecan. (A) Sub-G1 analysis of wild type and cisplatin-resistant SW620 cells treated with 20 nM topotecan for 72 h. Values are the mean (±standard deviation of the mean) of three independent experiments. (B) Wild type and cisplatin-resistant SW620 cells were treated with 20 nM topotecan for 72 h. Immunoblot analysis using the indicated antibodies is shown. The samples are indicated above; proteins of interest are indicated on the left. "WILD" – wild type cells; "RES" – cells resistant to cisplatin. "Contr" – control sample; "top" – topotecan.

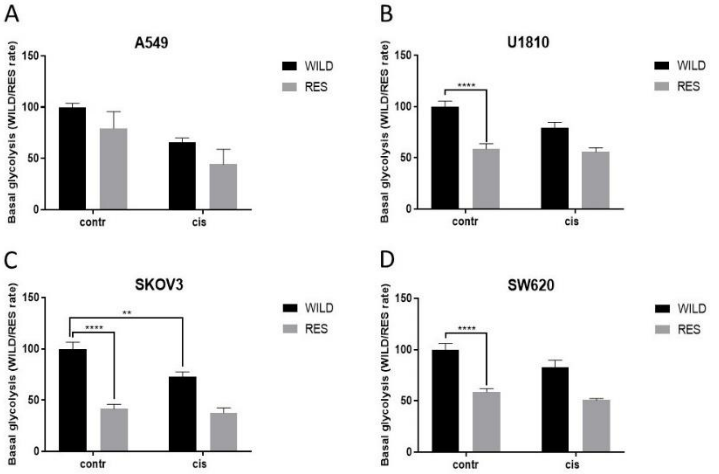

Figure S3. The assessment of basal glycolysis rate of A549 (A) and U1810 (B), SKOV3 (C), and SW620 (D) cell lines using the Seahorse XF Extracellular Flux Analyzer. The data were normalized to wild type cells and presented as WILD/RES rates (from 3 independent experiments). Two-way ANOVA with Sidak's multiple comparison test was used for statistical analysis. "WILD" – wild type cells; "RES" – cells resistant to cisplatin. "Contr" – control sample; "Cis" – cisplatin.

A

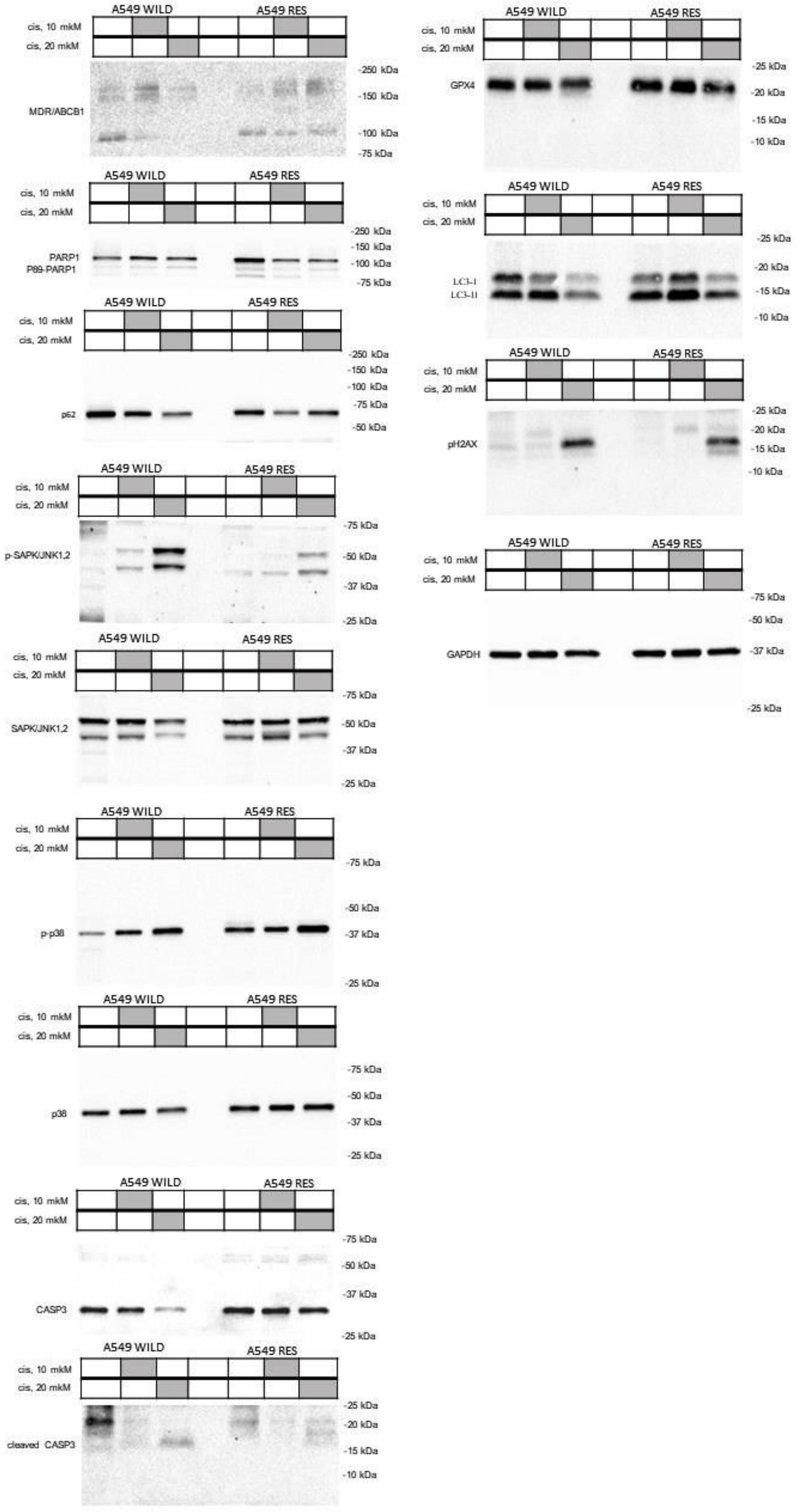
A549 WILD
A549 RES
cis, 10 mkM
cis, 20 mkM
MDR/ABCB1
PARP1
P89-PARP1
p62
p-SAPK/JNK1,2
SAPK/JNK1,2
p-p38
p38
CASP3
cleaved CASP3
GPX4
LC3-I
LC3-II
pH2AX
GAPDH
-250 kDa
-150 kDa
-100 kDa
-75 kDa
-50 kDa
-37 kDa
-25 kDa
-20 kDa
-15 kDa
-10 kDa

B

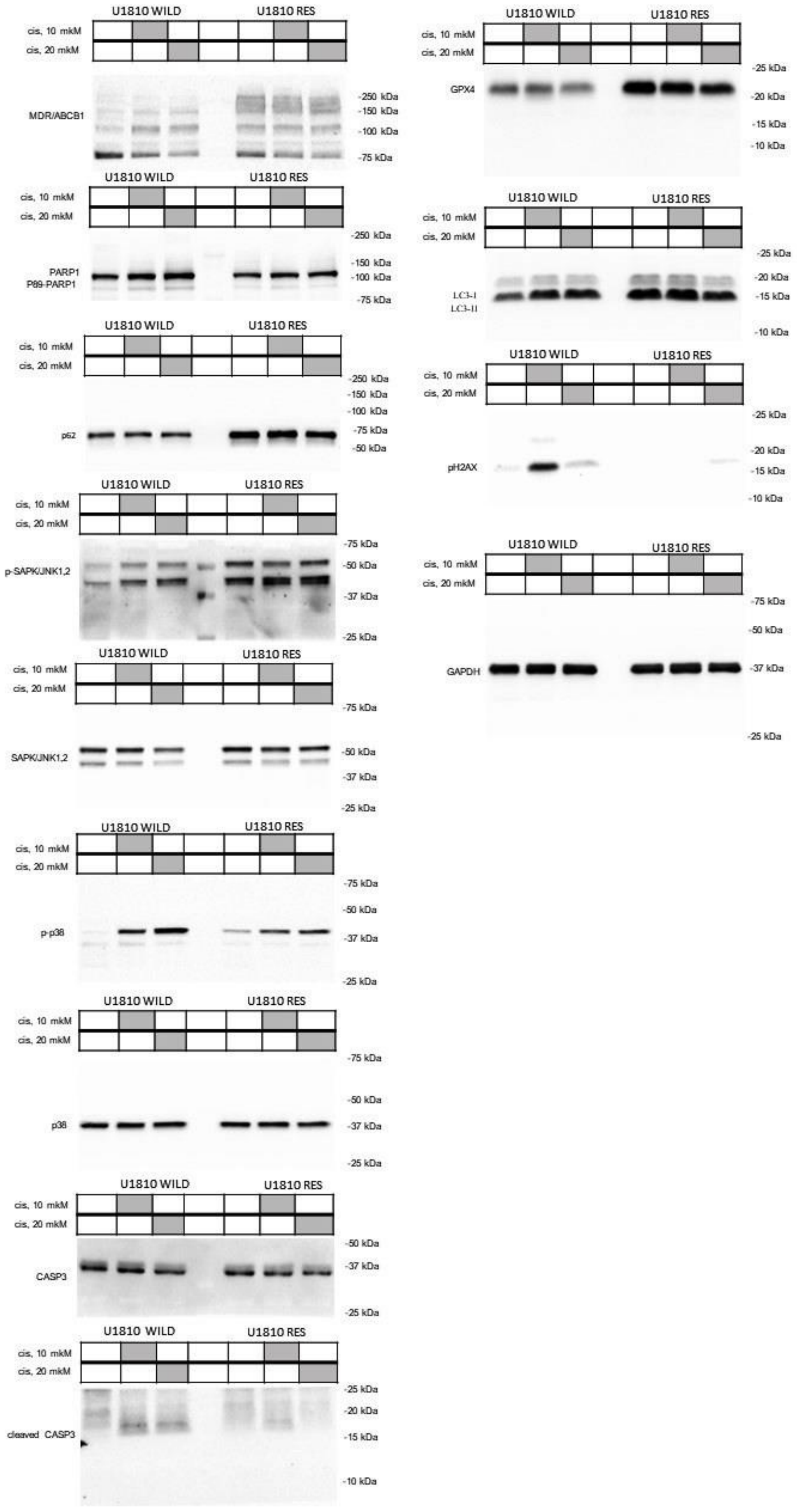
U1810 WILD
U1810 RES
cis, 10 mkM
cis, 20 mkM
MDR/ABCB1
-250 kDa
-150 kDa
-100 kDa
-75 kDa
PARP1
P89-PARP1
p62
-50 kDa
p-SAPK/JNK1,2
-37 kDa
-25 kDa
SAPK/JNK1,2
p-p38
p38
CASP3
cleaved CASP3
-20 kDa
-15 kDa
-10 kDa
GPX4
LC3-I
LC3-II
pH2AX
GAPDH

C

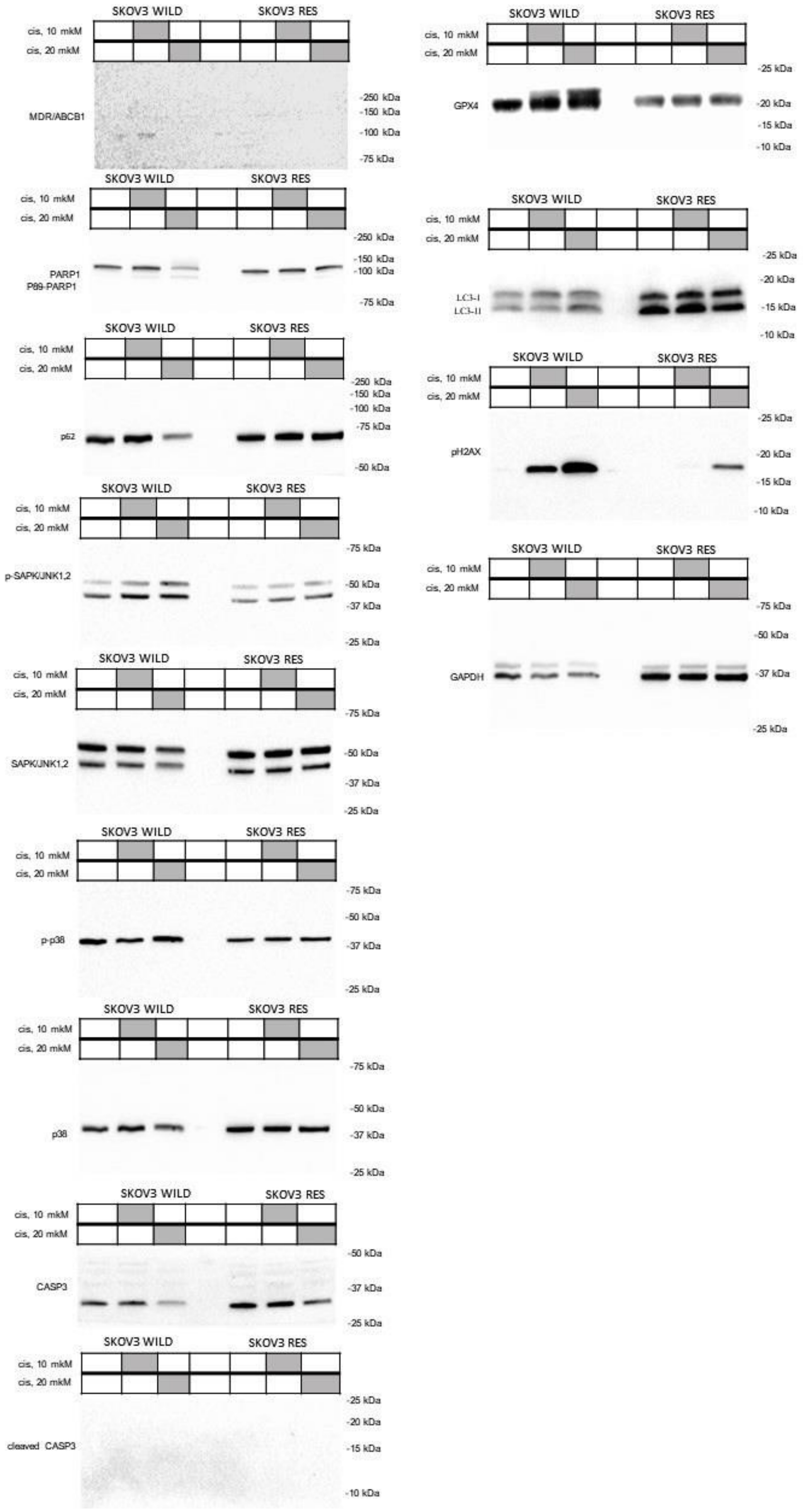
SKOV3 WILD
SKOV3 RES
cis, 10 mkM
cis, 20 mkM
MDR/ABCB1
-250 kDa
-150 kDa
-100 kDa
-75 kDa
PARP1
P89-PARP1
p62
-50 kDa
p-SAPK/JNK1,2
-37 kDa
-25 kDa
SAPK/JNK1,2
p-p38
p38
CASP3
cleaved CASP3
-20 kDa
-15 kDa
-10 kDa
GPX4
LC3-I
LC3-II
pH2AX
GAPDH

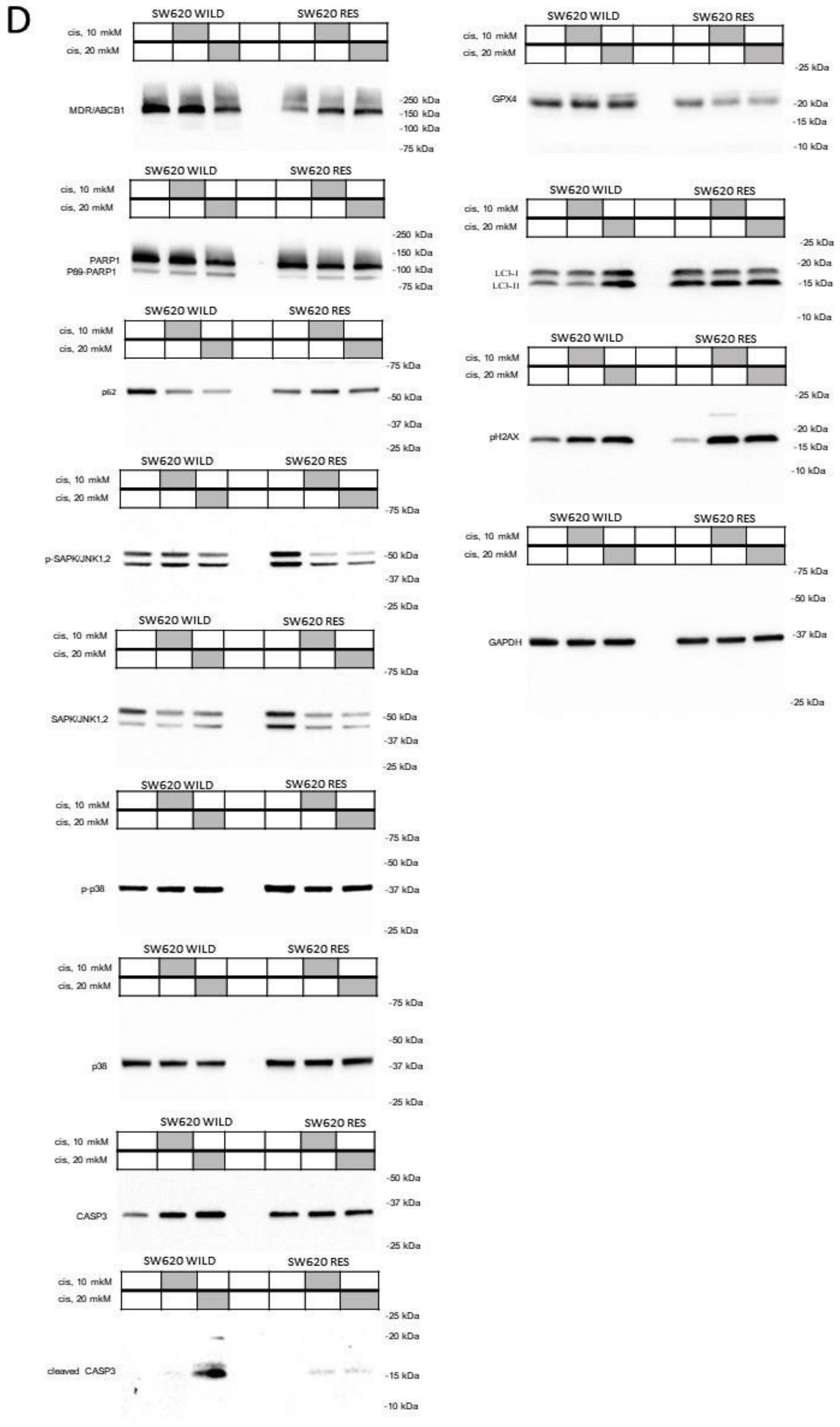

Figure S4. Uncropped immunoblot images of A549 (A) and U1810 (B), SKOV3 (C), and SW620 (D) cell lines.
